# HMGB1 Translocation in Neurons after Ischemic Insult: Subcellular Localization in Mitochondria and Peroxisomes

**DOI:** 10.3390/cells9030643

**Published:** 2020-03-06

**Authors:** Dengli Wang, Keyue Liu, Yusuke Fukuyasu, Kiyoshi Teshigawara, Li Fu, Hidenori Wake, Aiji Ohtsuka, Masahiro Nishibori

**Affiliations:** 1 Department of Pharmacology, Graduate School of Medicine, Dentistry, and Pharmaceutical Sciences, Okayama University, Okayama 7008558, Japan; dengliwang@okayama-u.ac.jp (D.W.); liukeyue@md.okayama-u.ac.jp (K.L.); yufukuyu@gmail.com (Y.F.); teshi@cc.okayama-u.ac.jp (K.T.); alisonfu@ust.hk (L.F.); wake-h@cc.okayama-u.ac.jp (H.W.); 2 Department of Human Morphology, Graduate School of Medicine, Dentistry, and Pharmaceutical Sciences, Okayama University, Okayama 7008558, Japan; aiji@okayama-u.ac.jp

**Keywords:** middle cerebral artery occlusion, high-mobility group box 1, subcellular localization and subcellular organelle

## Abstract

High mobility group box-1 (HMGB1), a nonhistone chromatin DNA-binding protein, is released from neurons into the extracellular space under ischemic, hemorrhagic, and traumatic insults. However, the details of the time-dependent translocation of HMGB1 and the subcellular localization of HMGB1 through the release process in neurons remain unclear. In the present study, we examined the subcellular localization of HMGB1 during translocation of HMGB1 in the cytosolic compartment using a middle cerebral artery occlusion and reperfusion model in rats. Double immunofluorescence microscopy revealed that HMGB1 immunoreactivities were colocalized with MTCO1(mitochondrially encoded cytochrome c oxidase I), a marker of mitochondria, and catalase, a marker of peroxisomes, but not with Rab5/Rab7 (RAS-related GTP-binding protein), LC3A/B (microtubule-associated protein 1 light chain 3), KDEL (KDEL amino acid sequence), and LAMP1 (Lysosomal Associated Membrane Protein 1), which are endosome, phagosome, endoplasmic reticulum, and lysosome markers, respectively. Immunoelectron microscopy confirmed that immune-gold particles for HMGB1 were present inside the mitochondria and peroxisomes. Moreover, HMGB1 was found to be colocalized with Drp1 (Dynamin-related protein 1), which is involved in mitochondrial fission. These results revealed the specific subcellular localization of HMGB1 during its release process under ischemic conditions.

## 1. Introduction

High mobility group box-1 (HMGB1) is released from neurons during ischemic injury, traumatic injury, intracerebral hemorrhage, and spinal cord injury [1,2,3,4,5,6]. The translocation of HMGB1 in neurons and their subsequent release were demonstrated in primary culture after the insult of glucose deprivation and glutamate treatment [7,8]. Once released into the extracellular space, HMGB1 affects component cells of the blood–brain barrier (BBB), vascular endothelial cells, and pericytes leading to the leakage of plasma protein and edema formation [2]. In a study using an in vitro BBB system, it was reported that stimulation with recombinant HMGB1 increased the permeability of the Evans blue–albumin complex in association with morphological changes in pericytes and endothelial cells [2], suggesting that HMGB1 may disrupt the BBB by acting directly on component cells. In addition, HMGB1 appears to stimulate microglia while simultaneously up-regulating inflammation-related molecules such as iNOS (inducible nitric oxide synthase), IL-1β (interleukin 1β), and TNF-α (Tumor necrosis factor) [9]. The treatment of middle cerebral artery occlusion (MCAO) rats with anti-HMGB1 monoclonal antibody remarkably inhibited the translocation of HMGB1 in neurons and suppressed the BBB disruption and brain inflammation, resulting in marked diminution of infarct size [1,2]. Moreover, it was suggested that the translocation and release of HMGB1 were the furthest upstream among a cascade of events in the acute phase of brain ischemia and trauma [1,2,3]. Thus, the translocation and release of HMGB1 from neurons appears to be a crucial step for triggering the brain inflammatory responses.

The dynamics of HMGB1 within cells has been reported using monocytes/macrophages. Acetylation and translocation of HMGB1 may occur after the stimulation of monocytes/macrophages with LPS (Lipopolysaccharide) and TNF. It has been demonstrated that LPS-induced HMGB1 secretion by monocytes is mediated by lysosomal exocytosis [10]. Acetylation or phosphorylation of HMGB1 has been suggested as the reason for the secretion from macrophages or fibrocytes. Kawabata et al. [11] suggested that the balance of acetylation and deacetylation of HMGB1 is disrupted when the acetylation status changes after the stimulation of monocytes/macrophages. However, the exact mechanisms by which HMGB1 is translocated and the precise subcellular pathway of HMGB1 in neurons during the release process after ischemic insult or trauma are poorly understood.

In the present study, the subcellular localization of HMGB1 after ischemic insult in rats was studied to better understand the pathways of translocation and release of HMGB1 in neurons. Our results showed that HMGB1 is colocalized within mitochondria and peroxisomes, but not within endosomes, lysosomes, phagosomes, or endoplasmic reticulum in the brain of MCAO rats, which may provide a new basis for further clarifying the mechanism by which HMGB1 affects ischemic rats after 2 h MCAO.

## 2. Materials and Methods

### 2.1. Animals and Experimental Procedures

All experimental procedures were conducted in accordance with the Okayama University Guidelines for Animal Experiments and were approved by the university’s Committee on Animal Experimentation (approval no. OKU-2017281). Male Wistar rats (Charles River, Yokohama, Japan) weighing 250–300 g were used for all experiments. MCAO for 2 h and subsequent reperfusion were performed as described previously [1]. Briefly, the rats were initially anesthetized with 3% isoflurane and maintained with 1.5–2% isoflurane in a mixture of 50% N_2_O and 50% O_2_. A silicone-coated 4.0 nylon thread was delivered to occlude the root of the right middle cerebral artery and the tip of the thread was placed distally (18 mm) from the bifurcation of the internal and external carotid arteries. All rats showed paralysis of the contralateral limbs after recovery from anesthesia. Two hours after MCAO, the rats were anesthetized again and the nylon thread was withdrawn to restore the cerebral blood flow for reperfusion. The rectal temperature was maintained at 37 °C with a heating lamp throughout the surgery. Brain tissue was collected at indicated time intervals (2, 6, 12, 24 h, and 10 day) after reperfusion.

### 2.2. Immunohistochemical Staining

Paraffin-embedded brain sections were prepared as described in a previous study [2]. The deparaffinized brain sections were incubated with the rabbit anti-HMGB1 antibody (Abcam PLC, Cambridge, UK) or mouse anti-HMGB1 monoclonal antibody (R&D Systems, Minneapolis, MA, USA) in combination with mouse anti-MTCO1 (mitochondrially encoded cytochrome c oxidase I, a marker of mitochondria) antibody, rabbit anti-catalase (a marker of peroxisomes) antibody, rabbit anti-LAMP1 (Lysosomal Associated Membrane Protein 1, a marker of lysosome) antibody, mouse anti-KDEL (KDEL amino acid sequence, a marker of endoplasmic reticulum), rabbit anti-LC3A/B (microtubule-associated protein 1 light chain 3, a marker of phagosome) antibody, rabbit anti-Rab5 (RAS-related GTP-binding protein 5, a marker of early endosome) antibody, rabbit anti-Rab7 (RAS-related GTP-binding protein 7, a marker of late endosome) antibody, rabbit anti-Drp1 (Dynamin-related protein 1, a marker of mitochondrial fission) antibody, rabbit anti-iba1 (Ionized calcium-binding adaptor molecule 1, a marker of microglia) antibody, or rabbit anti-GFAP (Glial fibrillary acidic protein, a marker of astrocyte) antibody (all the antibodies were acquired from Abcam PLC). Then, the sections were incubated with secondary antibodies conjugated with Alexa-488 or Alexa-555 (Invitrogen, Tokyo, Japan). Finally, the sections were mounted by VECTASHIELD Hardset Mounting Medium (Vector Laboratories, Burlingame, CA, USA) and observed under LSM 780 confocal microscopic system (Carl Zeiss, Jena, Germany).

### 2.3. Transmission Electron Microscopic (TEM) Examination

At 12 h after reperfusion, MCAO rats were anesthetized with an intraperitoneal injection of sodium pentobarbital (50 mg/kg) and perfused with 100 mL of saline followed by 100 mL of 4% paraformaldehyde and 2.5% glutaraldehyde in 0.1 mol/L cacodylic acid buffer (pH 7.3). The brain was carefully removed and immersed in the same fixative, and 2 mm coronal slices were prepared. The fixed brain was embedded in epoxy resin and cut into ultrathin sections. The sections were mounted on copper grids and droplets of blocking solution (1% goat serum and 1% bovine serum albumin in TBST) (Tris Buffered Saline with Tween 20) for 20 min. The sections were then incubated with anti-HMGB1 and anti-MTCO1 or anti-catalase primary antibody, respectively, for 2 days at 4 °C, followed by overnight incubation with secondary antibodies (10 or 50 nm gold secondary antibody diluted with blocking solution (1:50)) at 4 °C. After that, the sections were exposed to osmium tetroxide vapor for 1.5 h. Finally, all the sections were observed under a transmission electron microscope (H-7100; Hitachi, Tokyo, Japan).

### 2.4. Granule-Like Structures Count

Areas of interest (4 × 10^4^ μm^2^) were selected in the captured images of the cortex in the ipsilateral brain at the indicated time intervals after reperfusion of MCAO. The number of granule-like structures with the diameter more than 0.3 μm was counted in the cytosol in each cell at the indicated time intervals (six pictures were taken at each indicated time interval and 20 cells were counted in each picture). Granule-like structures greater than 0.6 μm in diameter were also counted. Then, the ratio of these two number values in each cell was calculated. For the measurement of mitochondrial length, images were obtained from each cortical section in contralateral and ipsilateral brain using an electron microscope. Six pictures were taken at 12 h after reperfusion. Thereafter, the individual mitochondrion length (long axis) was measured and the results were expressed as relative values of the contralateral side. Each experiment with counting was performed by investigators blinded to the treatment.

### 2.5. Statistical Analysis

For multiple comparisons, statistical significance was evaluated using one-way analysis of variance followed by Bonferroni posttest. To compare two groups, a Student *t* test was performed. A probability value of less than 0.05 was considered to be significant. The results were expressed as mean ± SD.

## 3. Results

### 3.1. HMGB1 Translocation in a Time-Dependent Manner in Neurons

**Figure 1:**
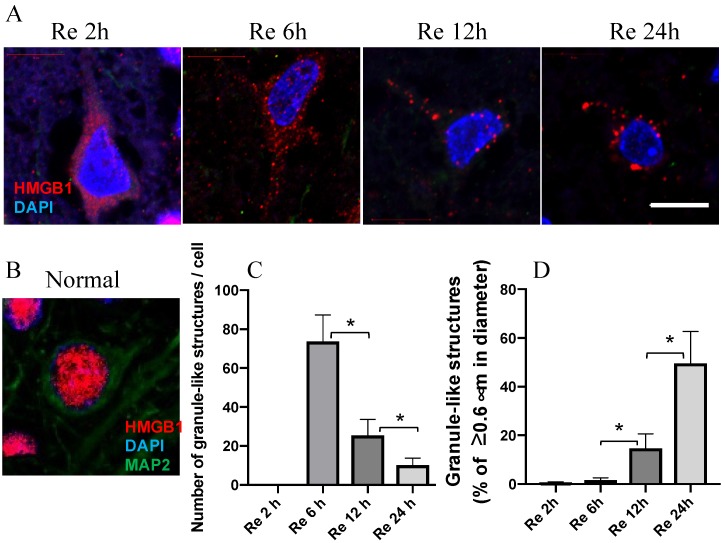
HMGB1 translocation at the indicated time intervals. (**A**) HMGB1 staining in a typical neuron in the parietal cortex at 2, 6, 12, and 24 h after reperfusion of MCAO. (**B**) HMGB1 staining in a typical neuron of cortex in normal rat brain. (**C**) The number of granule-like structures with the diameter more than 0.3 μm per cell in the cytosol at the indicated time intervals after reperfusion of MCAO. (**D**) The ratios of the number of granule-like structures with more than 0.6 μm in diameter vs. total number of granule-like structures in the cytosol were determined at the indicated time intervals after reperfusion of MCAO. Scale bar: 10 μm. (*n* = 6, * *p* < 0.05)

As reported in a previous study, the very early translocation of nuclear HMGB1 to the cytosolic compartment 2 h after reperfusion (Figure 1A) was observed in our study. The immunoreactivities of HMGB1 revealed granule-like structures in the cytoplasm in a time-dependent manner, particularly on the ipsilateral side. The granule-like structures on the cytosol of each neuron in the penumbra were counted at the indicated time intervals; the average numbers are presented in Figure 1C. The average numbers decreased time-dependently, whereas the size of the granule-like structures increased gradually in the cytoplasm from 2 to 24 h after reperfusion of MCAO (Figure 1A,C,D).

### 3.2. Colocalization of HMGB1 and MTCO1 and Catalase

**Figure 2:**
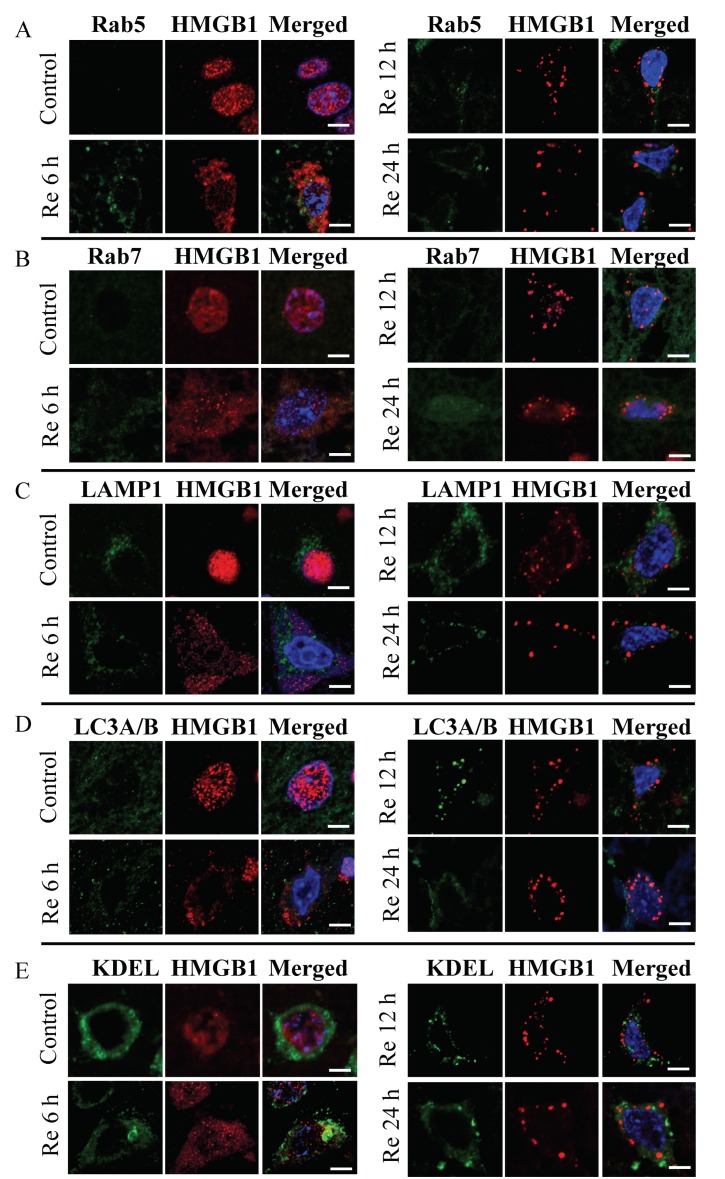
Immunostaining of HMGB1 with Rab5, Rab7, LAMP1, LC3A/B, and KDEL visualized with a confocal laser-scanning microscope. Coronal sections were prepared in control brain (sham group) and at 6,12, and 24 h after reperfusion. (**A**) Staining for HMGB1 (red) and Rab5 (green) was observed in the control brain, 6, 12, and 24 h after reperfusion. (**B**) Staining for HMGB1 (red) and Rab7 (green) was observed in the control brain, 6, 12, and 24 h after reperfusion. (**C**) Staining for HMGB1 (red) and LAMP1 (green) was observed in the control brain, 6, 12, and 24 h after reperfusion. (**D**) Staining for HMGB1 (red) and LC3A/B (green) was observed in the control brain, 6, 12, and 24 h after reperfusion. (**E**) Staining for HMGB1 (red) and KDEL (green) was observed in the control brain, 6, 12, and 24 h after reperfusion. Scale bar: 5 μm.

**Figure 3:**
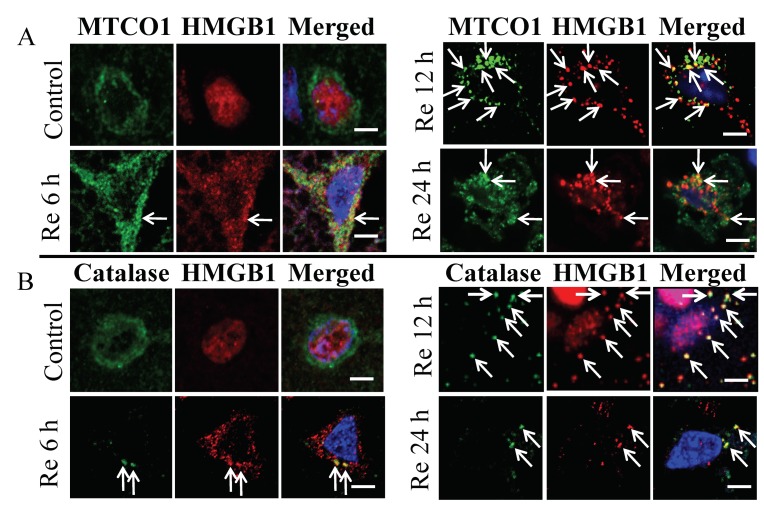
Immunolocalization of HMGB1, MTCO1, and catalase under a confocal laser-scanning microscope. (**A**) MTCO1 and HMGB1 shown in control brain (sham group), and 6, 12, and 24 h after reperfusion of MCAO. Merged images (yellowish color, white arrows) showing colocalization of HMGB1 in the mitochondria at 6, 12, and 24 h after reperfusion of MCAO. (**B**) Catalase and HMGB1 shown in control brain and 6, 12, and 24 h after reperfusion of MCAO. Merged images (yellowish color, white arrows) showing colocalization of HMGB1 in the peroxisome at 6, 12, and 24 h after reperfusion of MCAO. Scale bar: 5 μm.

To clarify the specific localization of these HMGB1 granule-like structures in the subcellular organelles, the brain sections were first treated with specific marker proteins for lysosomes, endosomes, phagosomes, endoplasmic reticulum, peroxisomes, and mitochondria, and then examined under a laser confocal microscope. Among the subcellular organelles, there was no merged image of HMGB1 on Rab5, Rab7, LAMP1, LC3A/B, and KDEL indicating that HMGB1 was not imported into the endosomes, phagosomes, lysosome, or endoplasmic reticulum in neurons at 6, 12, and 24 h after ischemia (Figure 2). In contrast, a merged image of HMGB1 immunoreactivities with those of mitochondria and peroxisomes (Figure 3A,B) was observed. As shown in Figure 3A,B, HMGB1 almost disappeared from the nuclei and a large amount of HMGB1 accumulated in the cytoplasm, which was partially colocalized with the mitochondrial marker MTCO1 and peroxisomal marker catalase in the ipsilateral cortex at 6, 12, and 24 h after ischemia.

### 3.3. Relationship of HMGB1 with MTCO1 and Catalase

**Figure 4:**
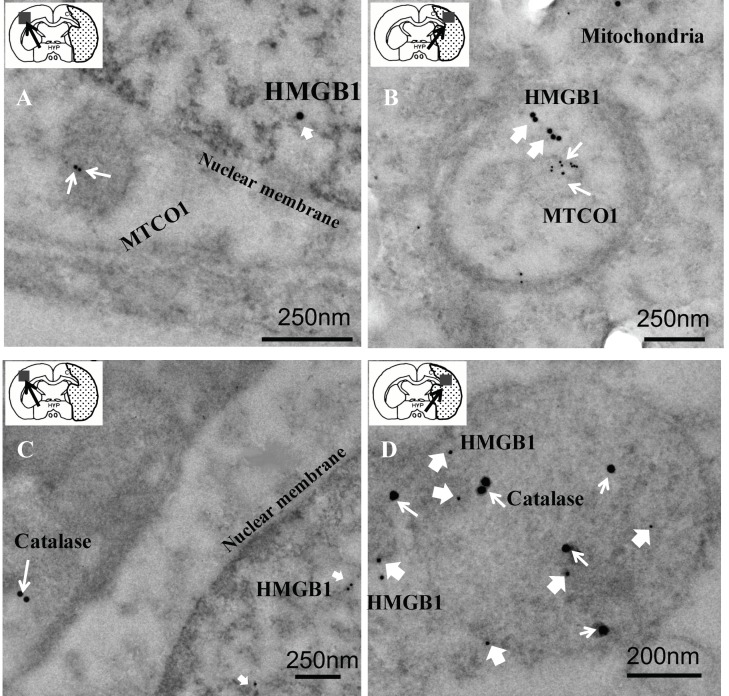
Electron microscopic immunogold localization studies of HMGB1 and MTCO1 or HMGB1 and catalase in the contralateral or ipsilateral hemisphere portion of the brain tissue sample at 12 h after reperfusion. (**A**,**B**) The anti-HMGB1 gold particles (20 nm, thick white arrows) or anti-MTCO1 gold particles (10 nm, thin white arrows) in a representative neuron in the contralateral (scale bar: 250 nm) or ipsilateral (scale bar: 250 nm) hemisphere portion of the brain tissue sample at 12 h after reperfusion, respectively. (**C**,**D**) HMGB1 localization visualized using 10 nm gold particles (thick white arrows) and catalase localization visualized using 20 nm (thin white arrows) gold particles in the contralateral (scale bar: 250 nm) or ipsilateral (scale bar: 200 nm) hemisphere portion of the brain tissue sample at 12 h after reperfusion, respectively.

To further elucidate the colocalization and examine the submitochondrial localization of HMGB1-positive spots more closely, immunoelectron microscopy was performed on ultrathin brain sections at 12 h after reperfusion of MCAO. In the contralateral region, HMGB1 particles were observed exclusively in the nucleus, and no specific labeling of HMGB1 was observed in the mitochondria of neurons (Figure 4A). In contrast, neuronal HMGB1 was relocalized to MTCO1-labeled mitochondria in the ipsilateral brain cells (Figure 4B). Electron microscopy also revealed that HMGB1 particles were present within the catalase-positive peroxisomes in ipsilateral brain cells 12 h after reperfusion of MCAO (Figure 4D).

### 3.4. Morphologies of Mitochondria Related to Mitochondrial Fission after MCAO

**Figure 5:**
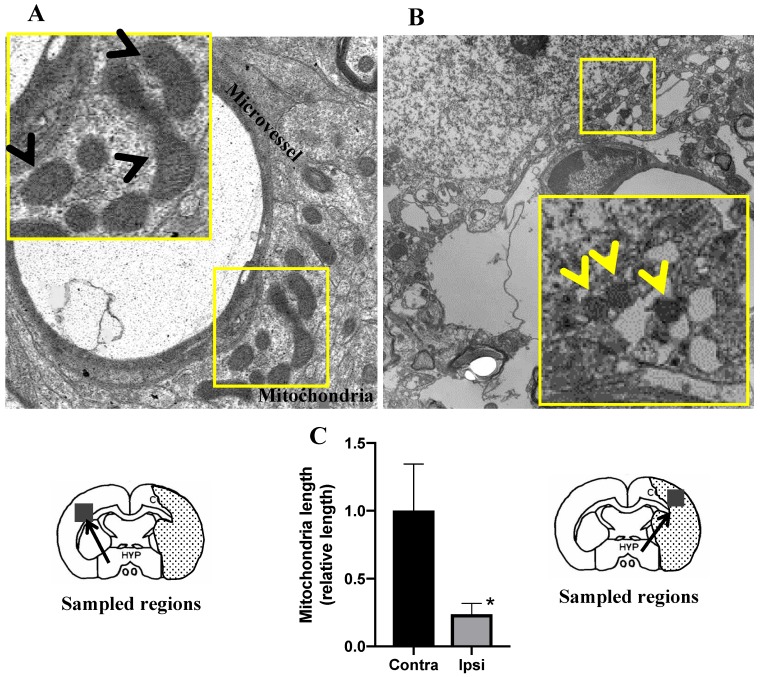
Brain microvessel with surrounding mitochondria visualized using TEM at 12 h after reperfusion. (**A**) A representative TEM image from the contralateral hemisphere. A higher magnification of the boxed area is shown at the upper left corner. Black arrow heads indicate normal mitochondria. (**B**) A representative TEM image from the ipsilateral hemisphere. A higher magnification of the boxed area is shown at the lower right corner. Yellow arrow heads indicate small and fragmented mitochondria. (**C**) The length of mitochondria in the contralateral and ipsilateral hemisphere were determined. The results were expressed as relative values of contralateral side. (*n* = 6, * *p* < 0.05).

**Figure 6:**
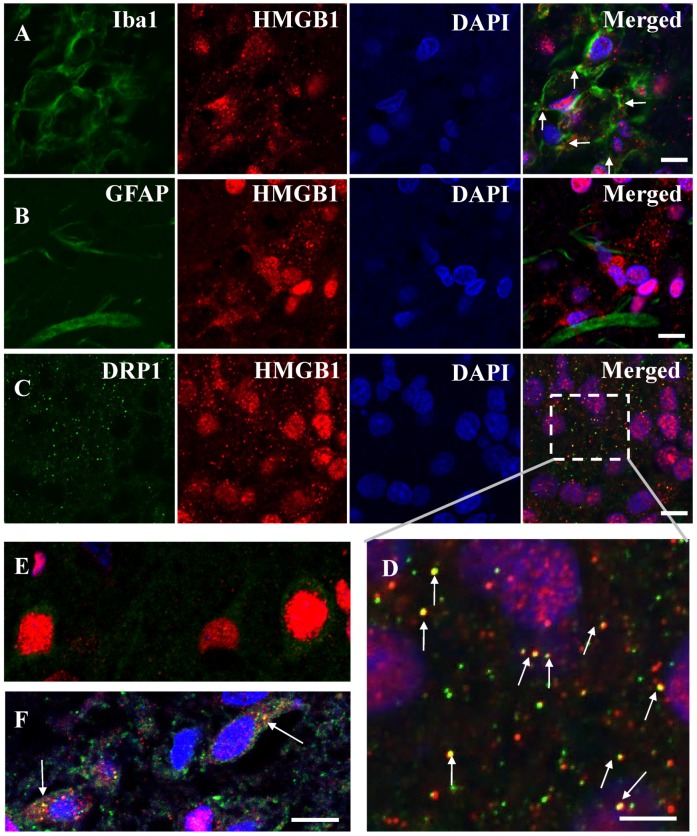
Immunostaining of HMGB1 with Iba1, GFAP, and Drp1. (**A**) Iba1 and HMGB1 staining at 10 days post-MCAO. (**B**) GFAP and HMGB1 staining at 10 days post-MCAO. (**C**) Drp1 and HMGB1 staining at 10 days post-MCAO. Scale bar: 10 μm. (**D**) Enlarged image of the boxed area in (C). Scale bar: 5 μm. (**E**) Drp1 and HMGB1 staining in the cortex of control brain (sham group). (**F**) Drp1 and HMGB1 staining at 12 h after reperfusion of MCAO. White arrows show the colocalization of HMGB1 and Drp1. Scale bar: 10 μm.

It is commonly believed that mitochondrial dysfunction plays a pivotal role in the pathophysiology of ischemia-reperfusion brain injury [12,13,14]. In this study, the morphologies of the mitochondria were assessed under TEM in ischemic brain cells. On the contralateral side, the morphology of mitochondria appeared mostly tubular. The mitochondrial matrices were evenly filled with electron-dense materials in the TEM image (Figure 5A). In contrast, the mitochondria in the ipsilateral region were small and fragmented (Figure 5B), and the length of the mitochondria was much shorter than that in the ipsilateral region (Figure 5C). Moreover, the loss of a normal cristae structure accompanied with many hollow areas was observed in the ipsilateral region at 12 h after reperfusion (Figure 5B), which suggested that the inner mitochondrial matrices were severely damaged. It is well known that mitochondrial fission is an early event required for ischemic neuronal death, and that ischemic neuronal death is mediated by Drp1, which cycles between the cytosol and the mitochondrial outer membrane [15]. To investigate the relationship between HMGB1 release and mitochondrial fission, double staining of HMGB1 and Drp1 after MCAO was performed. As expected, HMGB1 and Drp1 were merged in the cytoplasm of neurons at 12 h after reperfusion of MCAO (Figure 6F), which indicated that HMGB1 was translocated to small fragments formed by mitochondrial fission, suggesting that HMGB1 was released through the fragmented mitochondria and the translocation was related to mitochondrial fission. Interestingly, HMGB1 immunoreactivities were distributed in granules outside the microglial nuclei in addition to within nuclei in the core of the infarction area at 10 days post-MCAO (Figure 6A), suggesting that HMGB1 continued to be released from microglia in the core of the infarction area. These granules were not observed in astrocytes (Figure 6B). At 10 days after ischemia, Drp1 was colocalized with the translocated HMGB1 in microglia (Figure 6C,D).

## 4. Discussion

The neuronal translocation of HMGB1 has been examined by several groups after ischemic [1,2], traumatic [3], and epileptic injuries [16,17]. All these works clearly showed the translocation of HMGB1 from nuclei to the extracellular space through the cytosolic compartment. Although HMGB1 in the cytosolic compartment often showed the characteristic granular staining pattern, there is very limited information on the subcellular localization of HMGB1 in the cytosol. In the present study, the translocation of HMGB1 in neurons in the penumbral regions of the ischemic brain was confirmed. The typical pattern of the structure of the cytosolic localization showed a granule-like appearance, and this structure finally seemed to align along the cell membrane. Since the HMGB1 levels in the affected brain areas decreased significantly and the plasma and CSF levels of HMGB1 increased time-dependently in the previous studies [2], it was speculated that neuronal HMGB1 was secreted from neuronal nuclei to the extracellular space through the cytosolic compartment. Thus, the cytosolic localization of HMGB1 in the penumbral areas probably represents the intermediate phase of the secretory process in the ischemic brain.

It was demonstrated by double immunofluorescence staining that HMGB1 was colocalized with mitochondria and peroxisomes; these results were confirmed by the immunoelectron microscopic observations. The mitochondrial localization of HMGB1 might suggest the involvement of HMGB1 in the modulation of mitochondrial function under the ischemic condition. It was observed that the mitochondria became smaller and more round in the penumbral areas of MCAO rats at 12 h after reperfusion, which was consistent with the findings by Solenski et al. [14]. Severely damaged mitochondria may undergo fission to generate smaller spherical mitochondria. Drp1, which is present in the cytoplasm and binds to the mitochondrial outer membrane via Fis1, may play important roles in mitochondrial fission [18]. The synchronous colocalization of HMGB1 and Drp1 on mitochondria was observed under ischemic insult. The merged pictures of Drp1 with HMGB1 not only supported the colocalization of HMGB1 with Drp1, but also suggested the functional role of surface binding HMGB1 in mitochondrial fission. Interestingly, a recent study using a pilocarpine-induced status epilepticus model suggested that nuclear HMGB1 translocation and the subsequent mitochondrial import might deteriorate programmed necrotic neuronal death [19]. In addition, mitochondrial DNA (mtDNA) has been reported to be released from injured cells as DAMPs (damage associated molecular patterns), and HMGB1 has high affinity to DNA [20]. Thus, it might be possible that HMGB1 localization in the mitochondria had relevance to mtDNA release after ischemic insult. Moreover, the same study showed that HMGB1 activates the TLR9 signaling pathway by binding to released mtDNA to trigger inflammation and promote tumor growth in hepatocellular carcinoma during hypoxia [20]. In addition to mtDNA, other molecules, such as cytochrome c and mitochondria-derived reactive oxygen species, are also DAMPs from mitochondria, which might interact with HMGB1 in the inflammation and oxidative stress process during the mitochondria dysfunction in the ischemic brain. However, Ito et al. [21] observed that HMGB1 enhanced the repair of mtDNA by directly binding to mtDNA in spinocerebellar ataxia type 1 pathology, which indicated that HMGB1 might be relevant to the architectural control of mtDNA and probably promote neuronal regeneration and favor tissue repair through the process of mitochondrial fission/fusion in the later phase of ischemia. However, further works are necessary along this line.

In the case of peroxisomes, HMGB1-associated immunogold particles were present inside the peroxisomes. Among the subcellular organelles, little is known about the functional roles of peroxisomes except in metabolism, such as for the oxidation of fatty acid. The presence of HMGB1 in the peroxisomes strongly suggested that HMGB1 played another role in the functional regulation of cellular oxidative stress. Based on the specific localization of catalase, an H_2_O_2_-degrading enzyme, and other redox-related enzymes in peroxisomes, it has been speculated that peroxisomes are involved in the regulation of the redox state and a related intracellular signaling pathway [22,23]. Three cysteine residues on HMGB1 were reported to be sensitive to oxidative stress and to respectively form three kinds of molecular species: an all-thiol form, a disulfide form, and a sulfoxide form. Therefore, it might be possible that the occurrence of such transitions is dependent on the activity of peroxisome. Further studies will be needed to determine the molecular species of HMGB1 in the ischemic brain. In addition to the redox regulation, it has recently been suggested that peroxisomes function as pivotal regulators of immune functions and inflammation through immunometabolism [22]. It is worthy of mention that two HMGB1-localizing subcellular organelles, i.e., mitochondria and peroxisomes, might have a special interaction spatially and functionally [22,24]. Although a little is known about the regulatory role of HMGB1 in cell metabolism, the translocation of HMGB1 attached to peroxisomes during the ischemic responses in neurons may be related to the cellular metabolism.

Studies have suggested that HMGB1 undergoes multiple chemical modifications [25,26]. These include acetylation, methylation, phosphorylation, nitrosylation, oxidation, glycosylation, and ADP-ribosylation [25,27,28,29,30,31,32]. However, the relationship between the chemical modification and biological activity of HMGB1 remains largely unknown [33]. In macrophages or macrophage lineages, it was suggested that the release of HMGB1 occurred through a lysosomal pathway after acetylation of the HMGB1 molecule [25]. This was not applicable to neurons, because there were no merged pictures obtained after the double immunohistochemical staining with anti-HMGB1 and LAMP-1, a lysosome marker. In cancer cells, it has been suggested that HMGB1 may be involved in autophagy [34]. Thus, the subcellular secretory pathway for HMGB1 from the nuclei to extracellular space may vary depending on the cell types and stress conditions.

In the present study, dynamic changes in the size and number of HMGB1 granule like-structures in neurons from 2 to 24 h after reperfusion of MCAO were observed. This study also clearly demonstrated that HMGB1 was localized in mitochondria and peroxisomes in the cytosolic compartment of neurons in penumbral areas after ischemic insult in rats. In addition, it was shown that HMGB1 was colocalized with Drp1, which was involved in mitochondrial fission. Therefore, this typical localization suggested the involvement of HMGB1 in metabolic and inflammatory pathological processes in ischemic brain injury.
